# Comprehensive fluorescence profiles of contamination-prone foods applied to the design of microcontact-printed *in situ* functional oligonucleotide sensors

**DOI:** 10.1038/s41598-024-58698-0

**Published:** 2024-04-09

**Authors:** Shadman Khan, Amid Shakeri, Jonathan K. Monteiro, Simrun Tariq, Akansha Prasad, Jimmy Gu, Carlos D. M. Filipe, Yingfu Li, Tohid F. Didar

**Affiliations:** 1 School of Biomedical Engineering, McMaster University, 1280 Main Street West, Hamilton, ON L8S 4L8 Canada; 2 Department of Mechanical Engineering, McMaster University, 1280 Main Street West, Hamilton, ON L8S 4L7 Canada; 3 Department of Medicine, McMaster University, 1280 Main Street West, Hamilton, ON L8S 4K1 Canada; 4 Department of Biochemistry and Biomedical Sciences, McMaster University, 1280 Main Street West, Hamilton, ON L8S 4K1 Canada; 5 Department of Chemical Engineering, McMaster University, 1280 Main Street West, Hamilton, ON L8S 4L7 Canada

**Keywords:** Fluorescent dyes, Oligonucleotide probes, Fluorescence imaging, Food microbiology

## Abstract

With both foodborne illness and food spoilage detrimentally impacting human health and the economy, there is growing interest in the development of *in situ* sensors that offer real-time monitoring of food quality within enclosed food packages. While oligonucleotide-based fluorescent sensors have illustrated significant promise, the development of such on-food sensors requires consideration towards sensing-relevant fluorescence properties of target food products—information that has not yet been reported. To address this need, comprehensive fluorescence profiles for various contamination-prone food products are established in this study across several wavelengths and timepoints. The intensity of these food backgrounds is further contextualized to biomolecule-mediated sensing using overlaid fluorescent oligonucleotide arrays, which offer perspective towards the viability of distinct wavelengths and fluorophores for *in situ* food monitoring. Results show that biosensing in the Cyanine3 range is optimal for all tested foods, with the Cyanine5 range offering comparable performance with meat products specifically. Moreover, recognizing that mass fabrication of on-food sensors requires rapid and simple deposition of sensing agents onto packaging substrates, RNA-cleaving fluorescent nucleic acid probes are successfully deposited *via* microcontact printing for the first time. Direct incorporation onto food packaging yields cost-effective sensors with performance comparable to ones produced using conventional deposition strategies.

## Introduction

Despite extensive commercial and legislative efforts, foodborne illness caused by pathogenic contamination represents a growing global health concern, with an estimated annual caseload exceeding 600 million and over 420,000 associated deaths^1^. The associated cost of such illnesses continues to weigh heavily on healthcare systems, to the order of billions of dollars each year^2^. The economic effects of food contamination are further exaggerated by the associated food waste and the cost of retroactive food recalls, which are implemented once a contamination event is identified^3,4^. Alongside pathogenic contamination, food spoilage represents a concurrent concern. While illnesses caused by the consumption of spoiled food products offer significantly better prognoses, the predicted expiry dates used to prevent such events yield significant food waste due to their limited accuracy^5^. With several studies estimating that approximately a third of edible food products are wasted each year, continued societal reliance on static expiry dates has become a significant economic and environmental concern^6,7^.

Fueled by this crisis, the intersection between food science and biosensing has garnered significant interest. Efforts have been made towards the development of real-time, on-package contamination and spoilage detection platforms, which provide a comprehensive solution that would significantly improve food safety^8–11^. While various detection strategies have been applied in the development of on-package detection platforms, considerations toward commercial viability and ease-of-use have brought focus towards colorimetric and fluorescence transduction systems^9,12,13^. Colorimetric systems are optimal from an ease-of-use perspective, but the incorporation of colorimetric systems *in situ* has proven difficult. Specifically, most colorimetric signaling mechanisms require intricate reaction cascades that rely on numerous reagents^9^. The incorporation of such complex reaction mixes onto food packaging is largely impractical. While a few commercially feasible platforms have been proposed^11,14^, their real-world applicability is limited by their poor sensitivity and specificity.

Comparatively, fluorescence transduction platforms present a much more viable strategy, given the ease with which fluorophores can be integrated onto biorecognition molecules—such as protein and oligonucleotide probes, yielding singular entities with both recognition and reporting capabilities^15,16^. Antibodies have been somewhat explored within this space, however, oligonucleotide probes in particular, represent some of the most applied biorecognition probes, given their stability under varying conditions. These oligonucleotides have been labelled with fluorophores such as Pacific Blue, FITC, FAM, Cy3, and Cy5 to illicit fluorescent signals upon the identification of target molecules such as bacterial pathogens and chemical contaminants within food^17–19^. Yet, these oligonucleotides lack the functional properties required for food testing, making RNA-cleaving fluorescent nucleic acid probes (fNAPs) the most extensively explored platform in the food sensing space owing to their ease of functionalization, high stability, low cost, and simple detection cascade^20–24^. Composed of a probe strand joined to a substrate strand, their design usually involves the incorporation of a fluorophore-quencher pair within the substrate strand, flanking the ribonucleotide cleavage site. In the event of target exposure, cleavage induces the release of the quencher, resulting in fluorescence recovery^9^. With regards to contamination, several highly specific, pathogen-responsive fNAPs have been reported in recent years, some of which have shown efficacy within food-based applications^21,22^. Contrarily, fNAP-based approaches to spoilage detection have not been explored yet, but fluorescent aptamer-based systems targeting histamine—a spoilage marker for fish products such as tuna, have been reported^25^.

The development of *in situ* food sensors is expected to be a growing area of research, especially with the continued discovery of new probes that detect targets of interest with high sensitivity. However, with consideration towards subsequent sensor development, there is a lack of research aimed at determining relevant baseline properties of target food products—work that would better educate the design of future platforms. With regards to fluorescent sensors specifically, optimal transduction fluorophores for target food products are yet to be identified. Existing studies exploring the fluorescence spectra of food products have largely collected fluorescence profiles for compositional analysis and quality assessment^26^. While valuable, such works cannot inform the design of probe-based food sensors, as arbitrary quantifications of fluorescence cannot be readily compared to the intensity of fluorophore-labelled probes. Given the lack of data available regarding fluorophore visibility on food backgrounds, food sensing studies have employed a range of different fluorophores with no consideration towards the label best suited for their system, possibly limiting the resultant platforms’ sensitivity and reliability^27^.

Concurrently, growing interest in *in situ* food sensors also brings focus towards the ease with which sensing platforms can be integrated onto food packaging. While biomolecular microarrays are most often produced *via* non-contact piezoelectric deposition owing to significant commercial optimization, such systems remain slow^28^. As such, they are unsuitable for applications that require deposition over large surface areas, as would be required in the mass production of biofunctionalized food packaging^29^. Comparatively, contact printing can deposit target bio-inks over large substrates rapidly *via* roll-to-roll printing^29^. Further, the physical nature of contact deposition eliminates bio-ink evaporation post-deposition, inducing high lateral resolution within printed arrays^30^. Importantly, while the contact printing of antibodies has been successfully demonstrated in previous works^31–33^, the contact-based deposition of fNAPs has not yet been reported. Considering that contact printing offers limited control over biomolecule orientation—a necessary condition to permit signal-inducing fluorophore-quencher separation within fNAPs, developing functional arrays using such an approach can be considered difficult.

The presented work aimed to provide: (1) a comprehensive assessment of the inherent fluorescence background of several target food products across several wavelengths, (2) an evaluation of fluorophore-labelled oligonucleotide visibility when positioned alongside target food backgrounds, and (3) the first report of fNAP contact printing. Collectively, objectives 1 and 2 sought to deliver a holistic evaluation of the suitability of specific wavelengths and their associated fluorophores for *in situ* food monitoring—results that have high applicability towards the future design of sensors in this space. They also informed the design of probes used in objective 3, where contact-printed sensors directly embedded onto food packaging offered performance comparable to conventional array fabrication strategies, substantiating the presented approach.

## Results and discussion

**Figure 1:**
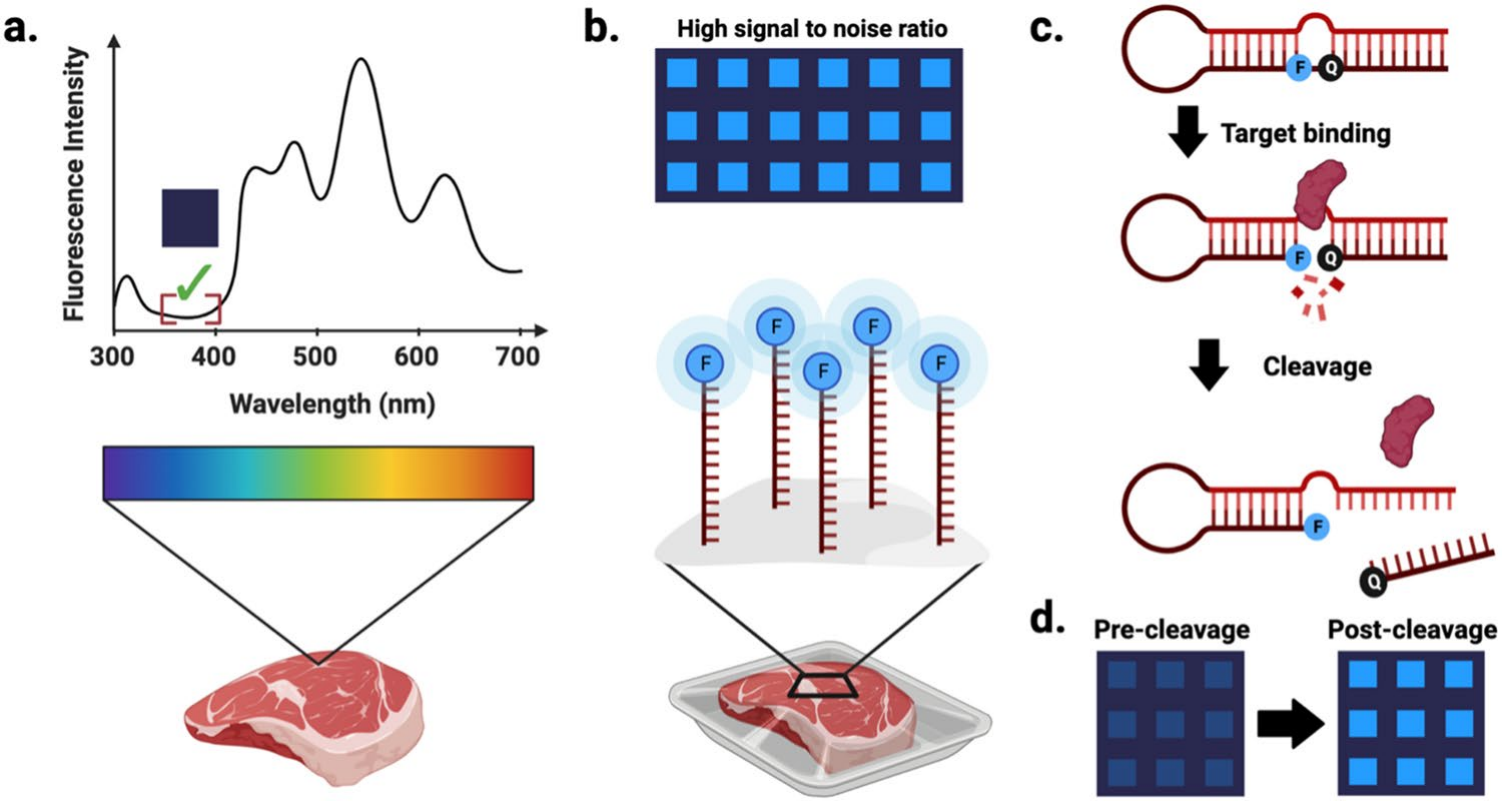
Schematic illustration of experimental approach. (**a**) Establishing inherent fluorescence profiles of target food products. (**b**) Evaluation of array visibility using fluorophore-conjugated oligonucleotides to identify labels that offer high signal to noise ratios. (**c**) Mechanism of action of RNA-cleaving fluorescent nucleic acid probes. (**d**) Fluorescence shift in fNAP microarrays following target-mediated fNAP cleavage. Created using BioRender.

Our experimental approach is summarized in Figure 1. Briefly, fluorescence profiles were derived for lettuce, spinach, chicken, and beef products through evaluation at four wavelengths commonly employed within fluorescence biosensing (Figure 1a). Fluorescence was also assessed over time to account for changes that occurred over the food products’ lifespans. Fluorophore visibility against target food backgrounds was evaluated using contact-printed oligonucleotides labelled with fluorophores corresponding to each of the four selected wavelengths (Figure 1b). Contact printed fNAPs were similarly immobilized onto food packaging, where they demonstrated both cleavage functionality and visibility with underlying food samples (Figure 1c,d).

### Establishing the fluorescence profiles of target food products

The inherent fluorescence of target food products was assessed using four channels with excitation/emission wavelengths of 350 nm/470 nm, 490 nm/525 nm, 557 nm/576 nm, and 649 nm/666 nm. Given that this collection of wavelengths extends throughout the entire visible light spectrum, the collected data comprehensively established the fluorescence profiles of the selected products. Further, these wavelengths roughly correspond with diamidino-2-phenylindole (DAPI), fluorescein isothiocyanate (FITC), tetramethylrhodamine (TRITC), and Cy5 fluorescence dyes, which yield blue, green, orange, and red emission, respectively^34–36^. Given that all commercially available fluorophores exhibit excitation/emission wavelengths comparable to one of these four dyes, the study was also comprehensive from a fluorescence biosensing perspective.

**Figure 2:**
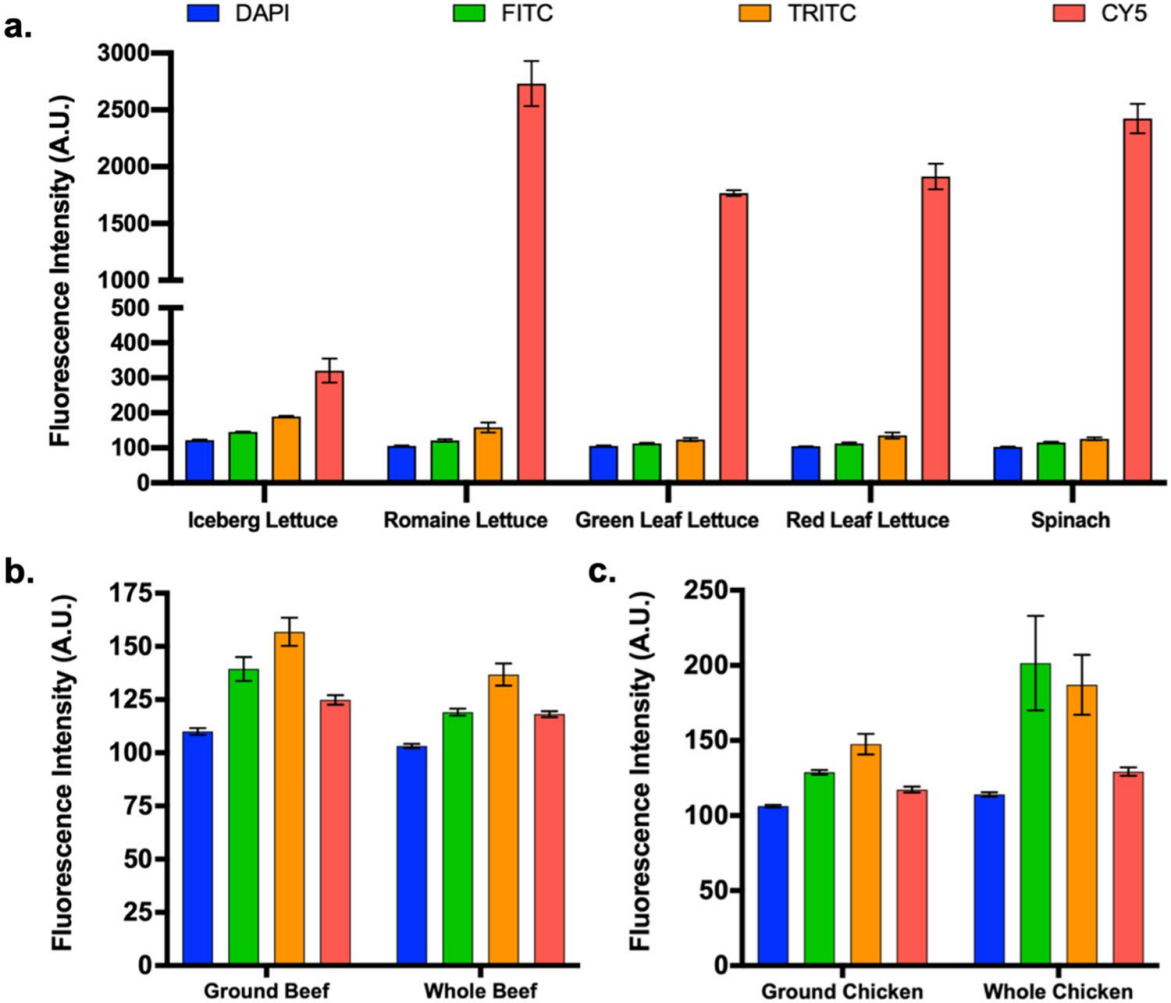
Baseline fluorescence properties of target food products. (**a**) Mean fluorescence intensities of produce products. (**b** and **c**) Mean fluorescence intensities of beef and chicken samples, respectively. All values consist of at least four data points. Error bars and dashed lines represent standard deviation.

The five produce products selected for assessment were iceberg lettuce, romaine lettuce, green leaf lettuce, red leaf lettuce, and spinach (Figure 2a). Across DAPI, FITC, and TRITC fluorescence channels, the five products showed similar profiles. Specifically, sample fluorescence was low across all three channels, but increased gradually at higher wavelengths. Mean intensity values ranged from 102 to 122 arbitrary units (A.U.) in the DAPI channel, 113–146 A.U. in the FITC channel, and 124–190 A.U. in the TRITC channel. Comparatively, the Cy5 channel yielded significantly higher fluorescence. Here, iceberg lettuce samples exhibited a mean fluorescence intensity of 321 A.U. This was still much lower than the four other tested products, which exhibited mean intensity values in the range of 1768–2762 A.U. High Cy5 fluorescence was attributed to chlorophyll molecules, which are highly abundant within produce and emit red light^37^. Importantly, chlorophyll is significantly less prominent within iceberg lettuce, explaining its lower fluorescence intensity in the Cy5 channel^38^.

Next, recognizing that chlorophyll exhibits poor photostability^39^, we sought to assess whether the Cy5 fluorescence intensity of lettuce samples could be decreased *via* concentrated light exposure. Romaine lettuce samples were imaged over the course of four minutes, during which a significant reduction in fluorescence intensity was observed (Fig. S1). This reduction was characterized by a sharp initial decrease in fluorescence, followed by a more gradual downward slope, that collectively induced a reduction in mean intensity from 2454 A.U. to 1148 A.U. Yet, despite this significant reduction, fluorescence intensity in the Cy5 channel remained significantly higher than what was observed in the DAPI, FITC, and TRITC channels. As such, Cy5 labelling was noted as unsuitable for produce-targeting biosensing applications.

With regards to meat products, beef and chicken samples were assessed (Figure 2b,c). Both ground and whole meat samples were evaluated to account for changes in fluorescence induced by post-slaughter processing. With ground beef samples, mean intensities of 110 A.U., 139 A.U., 157 A.U., and 125 A.U. were observed in DAPI, FITC, TRITC, and Cy5 channels, respectively. Comparatively, whole beef samples exhibited slightly lower mean fluorescence intensities, with values of 103 A.U., 119 A.U., 137 A.U., and 118 A.U. for each of the four channels, respectively. Ground chicken exhibited very similar mean intensities of 106 A.U., 129 A.U., 148 A.U., and 117 A.U. for DAPI, FITC, TRITC, and CY5, respectively. Whole chicken showed slightly higher mean fluorescence intensities of 114 A.U., 202 A.U., 187 A.U., and 129 A.U. for each of the four channels, respectively. Overarchingly, the relatively low intensities observed with meat samples across all evaluated channels yielded confidence towards broad applicability for biosensing.

### Monitoring changes in fluorescence profiles over product lifespans

Given that the visibility of signals from fluorescence sensors embedded within food packaging needs to be maintained throughout the course of a given food product’s lifespan, we then sought to evaluate changes in fluorescence over time. An optimal fluorescence channel would exhibit minimal changes over time, as such increases or decreases in background would make the calibration of signal-to-noise sensing thresholds unfeasible. Fluorescence consistency was quantified by the range of intensity values observed over the course of a 10-day test period.

**Figure 3:**
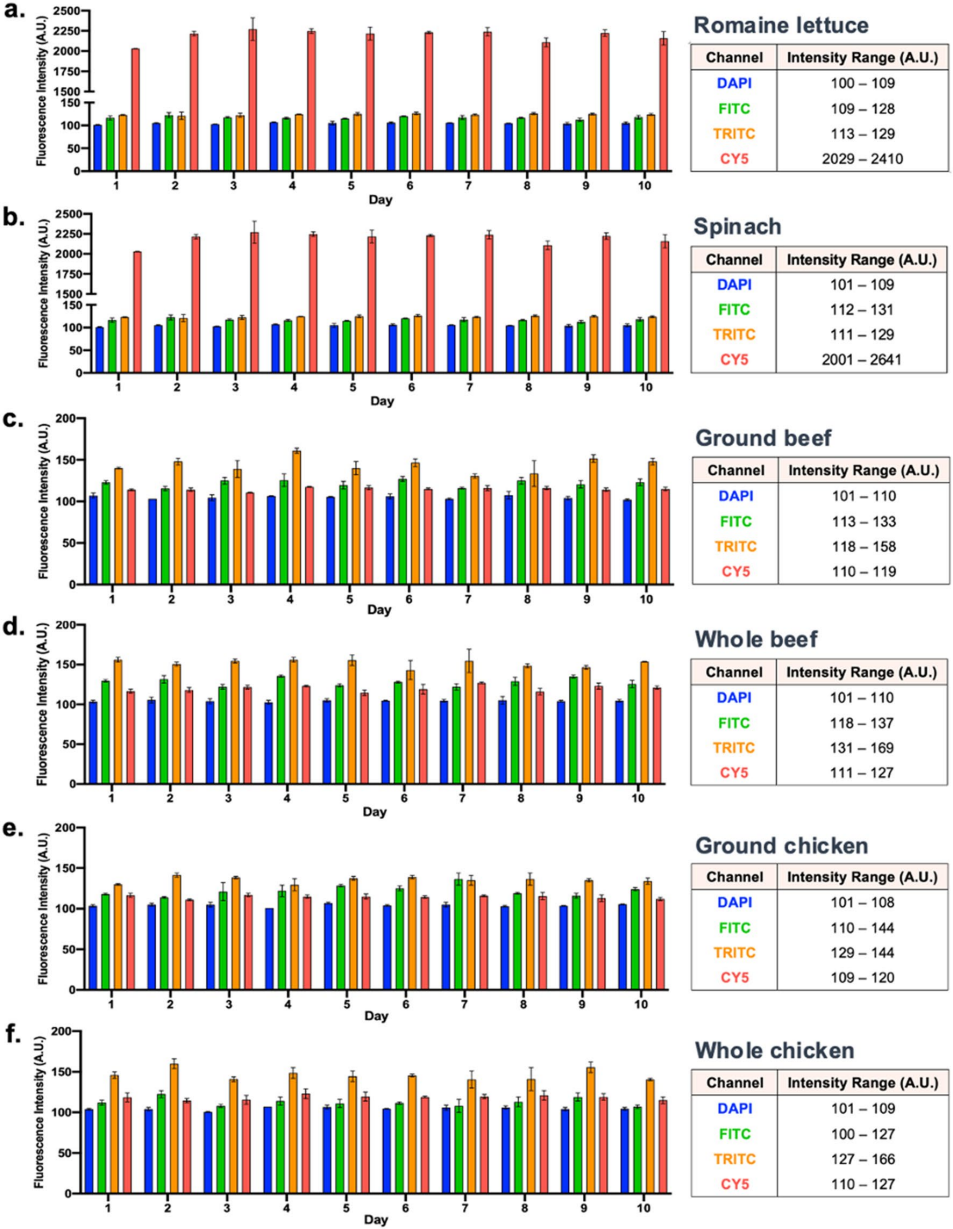
Changes in mean fluorescence intensity of target food products. Graphical depiction paired with intensity ranges for each evaluated wavelength provided for (**a**) romaine lettuce, (**b**) spinach, (**c**) ground beef, (**d**) whole beef, (**e**) ground chicken, and (**f**) whole chicken. All values consist of at least four data points. Error bars represent standard deviation.

With regards to produce, romaine lettuce and spinach samples were assessed, with limited variations in fluorescence intensity being observed in the DAPI, FITC, and TRITC channels (Figure 3a,b). Specifically, the range between the minimum and maximum fluorescence intensity values obtained for romaine lettuce were 9 A.U., 19 A.U., and 16 A.U. in the three channels, respectively. Similarly, spinach samples exhibited limited fluorescence variations, equating to ranges of 8 A.U., 19 A.U., and 18 A.U., respectively. Such high fluorescence consistency maintained the viability of these three channels for biosensing applications. Contrarily, large variations were observed in the Cy5 channel, with romaine lettuce and spinach exhibiting ranges of 381 A.U. and 640 A.U., respectively. This further highlighted Cy5 as unsuitable for produce.

In relation to beef samples, ground beef exhibited fluorescence ranges of 9 A.U., 20 A.U., 40 A.U., and 9 A.U. in the DAPI, FITC, TRITC, and Cy5 channels, respectively, while whole beef displayed ranges of 9 A.U., 19 A.U., 38 A.U., and 16 A.U (Figure 3c,d). Here, the higher fluorescence variation observed within the TRITC channel was noted as a potential hinderance to its use. Comparatively, ground chicken offered fluorescence ranges of 7 A.U., 34 A.U., 15 A.U., and 11 A.U. in the four respective channels, while whole chicken exhibited ranges of 8 A.U., 27 A.U., 39 A.U., and 17 A.U (Figure 3e,f). Accordingly, the FITC and TRITC channels were identified as wavelengths at which higher variation is present.

Collectively, DAPI offered limited fluorescence variation with each target food product, yielding confidence towards its applicability within sensing. Cy5 offered similarly low variation with meat products. While moderate variation was observed within TRITC and FITC channels, contextualizing the effect such variation would have on biosensing proved difficult given that the presented fluorescence intensities were quantified as arbitrary units. Thus, their value was limited to relative comparisons between channels and over time. To better understand how these background food intensities compare to what is emitted by fluorescently labelled biomolecules, we sought to assess the visibility of such agents when imaged against these products.

### Assessing fluorophore-biomolecule conjugate visibility on food backgrounds

Recognizing that many food monitoring systems employ oligonucleotide probes, we immobilized fluorophore-labelled, single-stranded DNA oligonucleotides onto transparent polyethylene food wraps, which were then imaged with target food products^23,40–42^. To this end, DNA oligonucleotides labelled with Pacific Blue, FAM, Cy3, and Cy5 fluorophores—compatible with DAPI, FITC, TRITC, and Cy5 channels, respectively, were deposited onto the polymer substrate *via* microcontact printing. Immobilization *via* physical adsorption was employed to eliminate the possibility of confounding fluorescence induced by crosslinking reagents.

**Figure 4:**
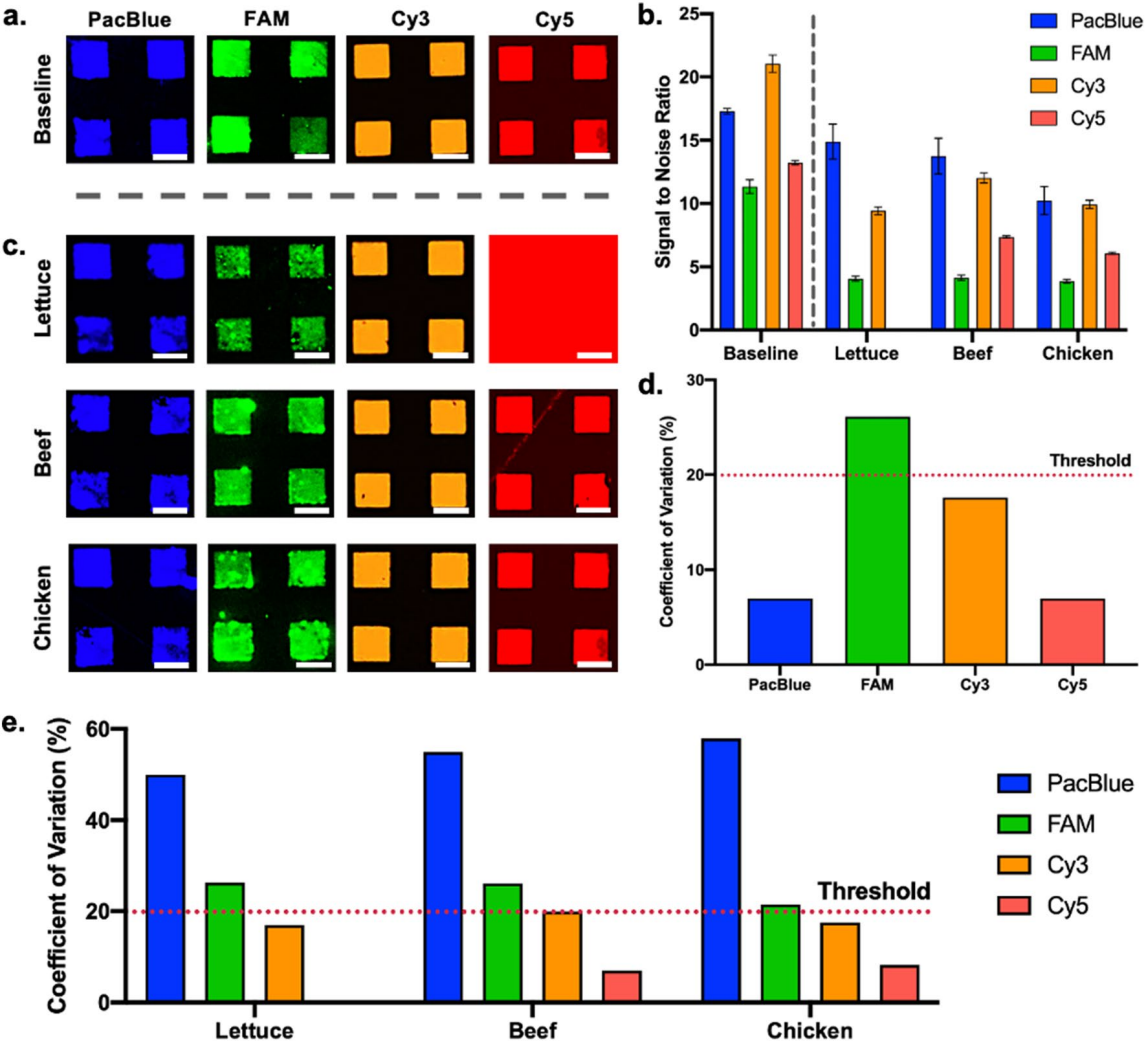
Visibility of contact-printed microarrays composed of fluorophore-labelled DNA oligonucleotides. (**a**) Fluorescence images of DNA microarrays deposited onto polyethylene food packaging. (**b**) SNR values of fluorescent DNA microarrays at baseline and with overlaid products. Each value consists of 30 data points. Error bars represent standard deviation. (**c**) Fluorescence images of DNA microarrays once overlaid with target food products. (**d**) Coefficient of variation of baseline DNA microarrays. Performance threshold identified with a dashed line. (**e**) Coefficient of variation of DNA microarrays overlaid on target food products. All scale bars depict 50 μm.

Each of the fluorophore-labelled DNA oligonucleotides formed high-resolution arrays (Figure 4a). As expected, differences in intensity were observed between the different fluorophores, which was attributed to the varying properties of each fluorescent label and their compatibility with contact printing. With consideration towards the baseline fluorescence noise produced by the polyethylene substrate, signal-to-noise ratios (SNR) were used to quantify the intensity of each array (Figure 4b). SNRs of 17.3, 11.3, 21.0, and 13.2 were derived for the Pacific Blue, FAM, Cy3, and Cy5 arrays, respectively. Subsequent visualization with overlaid food products employed romaine lettuce, ground beef, and whole chicken, given that all evaluated products showed fluorescence profiles comparable to one of these three products (Figure 4c). SNR values were used to quantify reductions in array visibility induced by food (Figure 4b).

Microcontact-printed arrays fabricated using the four respective fluorescence labels produced SNR values of 14.9, 4.1, 9.4, and 0, respectively when overlaid with romaine lettuce. This corresponded with 13.9%, 63.7%, 55.2%, and 100% reductions in SNR, respectively, relative to baseline SNR values. Accordingly, Pacific Blue was identified as an excellent candidate label, due to both its high baseline SNR and limited SNR reduction with romaine lettuce. Opposingly, the FAM-labelled oligonucleotide lost significant visibility—an outcome driven by both a low baseline SNR and significant romaine lettuce-induced fluorescence impedance. While the Cy3-labelled microarray also experienced significant fluorescence interference from overlaid romaine lettuce, high visibility was still retained, owing to a very high baseline SNR. Lastly, the Cy5 microarray was completely unobservable given the high fluorescence background produce exhibits at this wavelength.

Pacific Blue, FAM, Cy3, and Cy5 DNA microarrays imaged with underlying ground beef exhibited SNR values of 13.7, 4.2, 12.0, and 7.4, respectively. SNR was resultantly noted to have decreased by 20.8%, 62.8%, 42.9%, and 43.3% with the respective labels. Similar to romaine lettuce samples, the presence of a ground beef background slightly reduced the SNR of the Pacific Blue-labelled microarray, but to a comparatively limited degree. The reduction in the visibility of the FAM-labelled DNA microarray when imaged with a beef background was identical to what was observed with produce—a finding substantiated by the similar fluorescence profiles both foods exhibit at this wavelength. Cy3-labelled microarrays also behaved similarly between the two products, with a high SNR being maintained despite fluorescence interference from the overlaid food sample. The most notable difference with beef samples was observed with the Cy5-labelled microarray, as the significantly lower background fluorescence of meat at this wavelength allowed for successful visualization of the printed arrays. Nonetheless, the Pacific Blue and Cy3-labelled DNA microarrays offered higher visibility, owing to the lower baseline SNR of the Cy5-labelled microarray. Finally, the four respective microarrays exhibited SNR values of 10.2, 3.9, 9.9, and 6.1, respectively, when overlaid with chicken samples. This correlated with SNR reductions of 41.0%, 65.5%, 52.9%, and 53.8% relative to the baseline arrays. Again, Pacific Blue and Cy3-labelled microarrays offered the highest SNR values, despite significant fluorescence interference from overlaid food samples. Similar to what was observed with beef, the FAM-labelled microarray offered considerably low visibility and the Cy5-labelled microarray offered moderate visibility.

As a secondary measure of performance, the variation observed within printed arrays was also considered. Coefficient of variation (CV) values were calculated from fluorescence images, with a performance threshold of <20% being implemented. Pacific Blue, Cy3, and Cy5-labelled baseline microarrays exhibited CV values of 7.0, 17.6, and 7.0, respectively—all within the acceptable range (Figure 4d). Comparatively, the CV value of FAM-labelled baseline microarrays was 26.1. Higher variation here was attributed to unintended transfer of PDMS residue onto the polyethylene substrate during micro-contact printing, which causes non-specific fluorescence at this wavelength^43^. Various approaches to minimize this transfer have been previously explored in literature, including plasma functionalization and sonication^44,45^.

When overlaid with foods, significant changes in CV values were observed (Figure 4e). Specifically, Pacific Blue-labelled microarrays exhibited very high CV values of 50.0, 55.0, and 57.9 with overlaid romaine lettuce, beef, and chicken, respectively. Thus, while these microarrays retained high SNR values when imaged with target foods—owing to their high baseline fluorescence intensity, their high variability limits their suitability for biosensing. Recognizing that Pacific Blue offers comparatively low stability^46^, it is likely that interactions with complex food matrices induced variable degradation of the label. FAM-labelled microarrays offered better consistency, but still surpassed the <20% performance threshold, with CV values of 26.3, 26.1, and 21.5 with the three respective food products. Opposingly, Cy3 and Cy5 offered values consistently below the set threshold, with Cy3 offering CV values of 16.9, 19.9, and 17.6 for the three respective products, and Cy5 exhibiting values of 7.0 and 8.2 for beef and chicken, respectively.

Based on all the presented data, Cy3 range fluorophores appears to offer the highest compatibility with the tested food products, based on the inherent fluorescence profiles of target products and the concurrent visibility of Cy3 arrays. While Pacific Blue-labelled microarrays offer high SNR values, poor CV values bring into question its stability, which limits its applicability for lengthy incubations with food. Cy5 range fluorophores exhibit excellent performance but are compatible only with meat products since the emission wavelength of chlorophyll is within the range of these fluorophores, rendering it incompatible with produce products^37–39^. Finally, FITC range fluorophores offered comparatively poor performance. While a SNR of about 4 was observed with overlaid food samples, substantiating its potential use within food monitoring, better alternatives are available.

### Stability and functionality of microcontact printed fNAPs

Recognizing that Cy3 range fluorophores appear best suited for *in situ* sensing applications targeting the selected food products, we aimed to investigate the visibility of fNAPs conjugated with such labels. To this end, an aminated 5-Carboxytetramethylrhodamine (TAMRA)-labelled fNAP construct was employed. While fluorescently labelled, single-stranded oligonucleotides can be visualized with relative ease given the abundance of fluorophores, fNAP visualization is complicated by the presence of associated quenchers. Confirming that such functional molecules can be visualized when exposed to food backgrounds is key in oligonucleotide-mediated sensing. Functionality was assessed using sodium hydroxide (NaOH), given its ability to hydrolyze the ribonucleotide cleavage site efficiently, thus inducing maximal fNAP signal generation.

**Figure 5:**
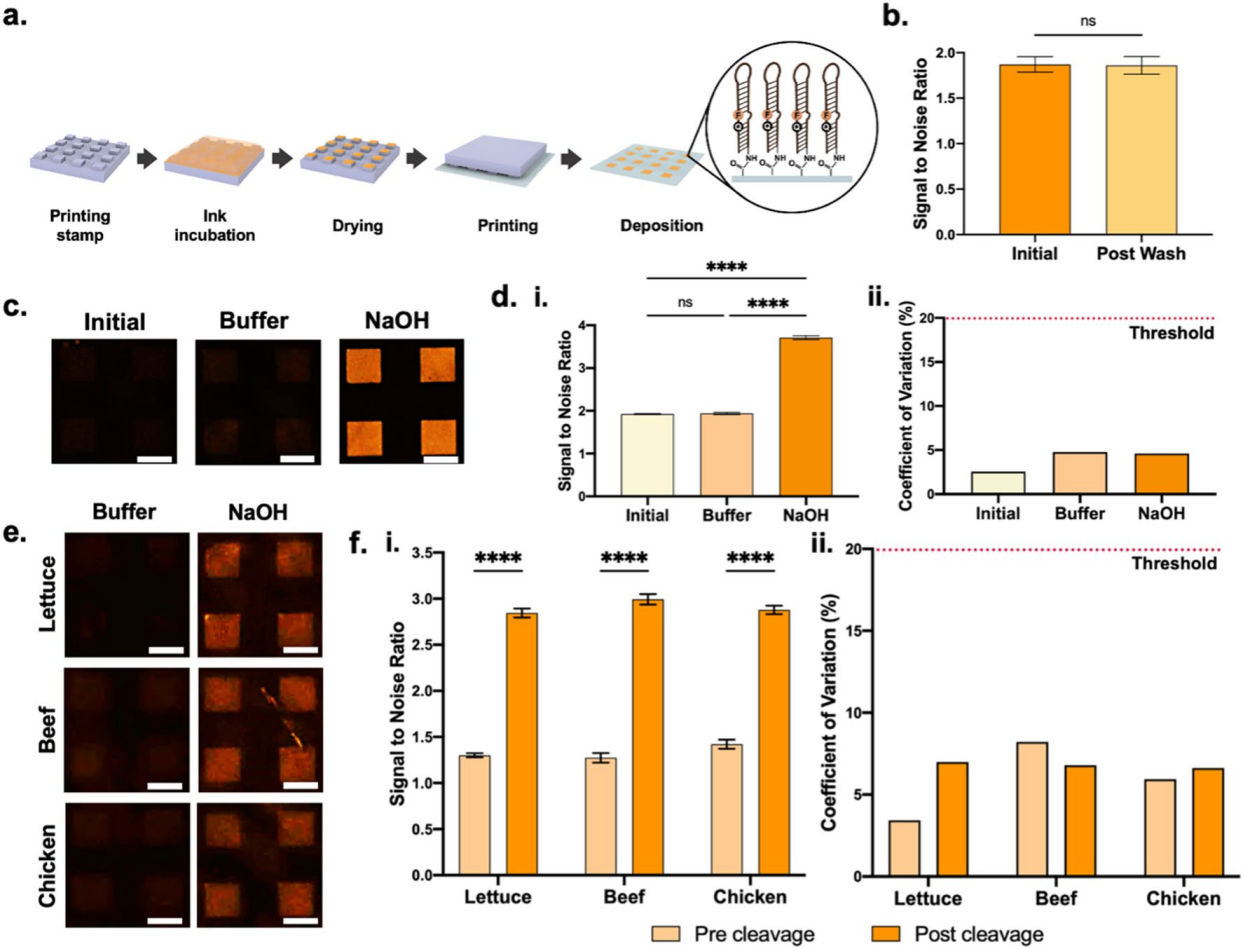
Stability and functionality of fNAP arrays deposited *via* microcontact printing. (**a**) Schematic illustration of fNAP microcontact printing. (**b**) SNRs of fNAP arrays before and after wash cycle. (**c**) Fluorescence images of fNAP arrays before and after testing. (**d**) (i) SNRs and (ii) CVs of fNAP arrays before and after testing. (**e**) Fluorescence images of fNAP arrays overlaid with target food products. (**f**) (i) SNRs and (ii) CVs of positive and negative state fNAP arrays when overlaid with target food. Error bars show standard deviation. Significance is indicated via ns/**** markers, corresponding to no significance and *P <* 0.0001, respectively. Scale bars show 50 μm.

A bio-ink composed of 3 μM fNAP solution and N-ethyl-N′-(3-(dimethylamino)propyl) carbodiimide/N-hydroxysuccinimide (EDC/NHS) crosslinker solution was used to induce covalent immobilization onto carbon dioxide plasma-treated polyethylene substrates. Yet, owing to the limited control previously reported protocols for microcontact printing offer over biomolecule orientation^32^, insignificant changes in fluorescence intensity were observed following NaOH exposure. Thus, to better mediate fNAP orientation we introduced two modifications to our protocol (Figure 5a). First, the volume of residual solution present on stamps prior to deposition was increased. While this reduced print quality at times, it increased bio-ink transfer onto the target substrate. Second, printing was performed under 65% humidity. Both these measures increased moisture in the printing environment, yielding increased moisture on the target substrate. We hypothesize that this moisture allowed for some degree of fNAP and crosslinker mobility post-deposition, thus facilitating proper crosslinking and fNAP orientation.

Following deposition, fNAP microarrays were exposed to a vigorous wash cycle to evaluate the stability of fNAP attachment (Figure 5b). Quantitative measurements demonstrated an insignificant degree of detachment, with the SNR remaining at 1.8. With microarray stability established, functionality tests using NaOH were conducted, where a magnesium chloride-rich buffer solution acted as the negative control. Resultant images are provided in Figure 5c. While the baseline fNAP microarray exhibited a SNR of 1.9, NaOH exposure yielded a significant increase as depicted by a SNR of 3.7 (Figure 5d.i). This corresponded with a fold change of 2 respective to the baseline fNAP array. Importantly, the buffer control exhibited no evident change, retaining a SNR of 1.9. The three conditions offered CV values of 2.6%, 4.8%, and 4.6%, respectively, confirming high array consistency (Figure 5d.ii).

Next, the visibility of buffer and NaOH-exposed fNAP microarrays was evaluated against target food backgrounds. The resultant fluorescence images are presented in Figure 5e. While the presence of overlaid food products expectedly interfered with the visibility of fNAP microarrays, both pre and post-cleavage states remained distinguishable. Of course, pre-cleavage microarrays offered minimal visibility, with SNRs of 1.3, 1.3, and 1.4 for romaine lettuce, beef, and chicken, respectively (Figure 5f.i). Comparatively, post-cleavage microarrays exhibited SNRs of 2.8, 3.0, and 2.9 with the three respective food products. With optimization of fNAP concentration and incubation conditions, the SNR of such systems can be expected to improve. More definitively, image processing would significantly increase the SNR of the presented platform, as the reported values were all derived from unprocessed images to provide a baseline evaluation of sensor signal intensity. All conditions offered CV values below 10%, confirming minimal food-mediated disruption of the arrays (Figure 5f.ii).

Finally, to substantiate the microcontact printing of fNAPs against conventional approaches, its performance was compared to that of inkjet-printed fNAP arrays. Baseline inkjet arrays offered a SNR of 1.6, while the buffer and NaOH conditions yielded SNRs of 1.8 and 3.8, respectively (Fig. S2). CV values were found to be well below the established 20% threshold as well. Here, the performance of the contact and non-contact printing strategies was similar. When overlaid with food, pre-cleavage inkjet arrays offered SNRs of 1.0, 0.8, and 0.8 with romaine lettuce, beef, and chicken, respectively (Fig. S3). Comparatively, post-cleavage SNR with the three respective overlaid food products were 2.1, 1.7, and 1.8. This was considerably lower than what was observed with microcontact-printed fNAP arrays overlaid with food, suggesting that inkjet-printed fNAP arrays suffered more signal interference from food backgrounds. This can be attributed to the comparatively low deposition homogeneity exhibited within singular inkjet-printed dots, which was likely induced by oligonucleotide aggregation within deposited droplets as a result of unintended evaporation. Nonetheless, the CV values were below the set threshold.

## Conclusion

With growing interest towards *in situ* food sensing platforms, establishing relevant baseline properties of target food products is of utmost importance. Optical sensing offers significant promise due to the ease with which such systems can be assessed in real-time throughout the entire food production pipeline, substantiating the need for optical evaluation of target foods. Accordingly, the comprehensive fluorescence profiles presented here for romaine lettuce, spinach, chicken, and beef and the adjacent visualization of these foods with fluorophore-labelled biomolecular arrays provides a foundation for future works in this space. With consideration towards all the presented data, Cy3 range fluorophores are best suited for platforms targeting the aforementioned food products, owing to the low inherent backgrounds of the products at this wavelength, paired with the high SNR and low CV of Cy3-labelled DNA microarrays. DAPI and FITC range fluorophores offer strong and moderate degrees of viability, respectively, while Cy5 range fluorophores are only applicable with livestock products.

Importantly, commercial manufacturing of *in situ* platforms requires rapid fabrication of sensors that are easily integrated onto food packaging materials. To this end, we demonstrate the functionalization of polyethylene food packaging films with fNAPs *via* contact printing. We are the first to report the contact deposition of functional DNA oligonucleotides—an approach that offers significant commercial potential, specifically with recent developments in roll-to-roll printing. Further, it overcomes the limited lateral resolution and bio-ink evaporation issues often associated with non-contact printing. Through the deposition of a range of bio-responsive oligonucleotides—appropriately labelled with suitable fluorophores, multiplex platforms can be easily produced using the strategies detailed in this study. Future studies based on this work may explore automating this contact printing process with fNAPs at an industrial scale to enable offering cost efficient, individualized product contamination monitoring^47^. Additionally, signal amplification strategies such as the incorporation of bacteriophage or lubricant infused surfaces which minimize noise may also be explored to optimize SNR^22,48,49^.

## Materials and methods

### Inherent fluorescence imaging of target food products

All evaluated food products used in this study were sourced from three different local grocery stores to increase generalizability (Walmart, Fortino’s, Food Basics; Hamilton, Canada). Iceberg lettuce, romaine lettuce, green leaf lettuce, and red leaf lettuce samples were layered to a thickness of 10mm and placed onto glass slides. Whole chicken and whole beef samples cut to a thickness of 10mm were similarly placed onto glass slides. 10mm thick ground chicken and ground beef samples were prepared using a doctor’s blade deposition strategy to ensure homogenous thickness. Samples were imaged using a Nikon Ti-2 inverted microscope (New York, United States) at excitation and emission wavelength pairings of 350 nm/470 nm, 490 nm/525 nm, 557 nm/576 nm, and 649 nm/666 nm. The selected wavelengths cover the majority of the electromagnetic spectrum which spans from around 300 nm to 700 nm, enabling comprehensive fluorescence profiling for the variety of food products examined in this work. All images were taken at an exposure time of 300ms. Mean intensity was calculated for each collected image, and a minimum of ten images were taken for each sample. Average intensities for each food product were derived.

### Photobleaching analysis

Romaine lettuce samples with a thickness of 10 mm were imaged using a Nikon Ti-2 inverted microscope under the Cy5 fluorescence channel (649 nm/666 nm) for four minutes. Images were automatically taken every 300 ms at an exposure time of 1s and the mean fluorescence intensity of the collected images were quantified. This procedure was repeated three times.

### Fluorescence imaging of target food products over time

On Day 1, the target food products—romaine lettuce, spinach, ground beef, whole beef, ground chicken and whole chicken, were aliquoted into 10 g portions, which were stored within sealed bags at 4 °C. Each day, three samples were deposited as 10 mm slices onto glass slides as previously described. Samples were imaged under the same parameters as the baseline studies—at excitation and emission wavelength pairings of 350 nm/470 nm, 490 nm/525 nm, 557 nm/576 nm, and 649 nm/666 nm using a Nikon Ti-2 inverted microscope set to an exposure time of 300 ms. Mean intensity was calculated for each collected image, and a minimum of ten images were taken for each sample. This process was repeated five times using food products from different grocers to ensure reproducibility in the reported trends.

### Contact printing stamp preparation

Polydimethylsiloxane SYLGARD 184 (Dow Corning, Michigan, United States) was prepared using a 10:1 weight ratio of base resin to curing agent. This mixture was stirred for 10 min and subsequently desiccated for 20 min. The uncured mixture was then poured onto a micro-patterned silicon wafer to a thickness of 1 cm. The polydimethylsiloxane was then heat cured at 145 °C over 15 mins, mediating pattern transfer from the wafer to the polymer^32^. The cured polymer was then cut into 1 cm × 1 cm × 1 cm cubes, which were used as stamps for microcontact printing.

### Oligonucleotide contact printing

10 μL oligonucleotide solutions (Integrated DNA Technologies, Iowa, United States; Millipore Sigma, Texas, United States) containing 3 μM DNA diluted in DNase-free water (Thermofisher Scientific, Ontario, Canada) were deposited onto micro-patterned polydimethylsiloxane stamps and incubated for 8 min. Concurrently, glass slides wrapped with the target polyethylene substrate (Thomas Scientific, New Jersey, United States) were oxygen plasma-treated for 3 mins (PlasmaEtch, Nevada, United States). Subsequently, the stamps were briefly rinsed in phosphate buffered saline (Bioshop Canada, Ontario, Canada). and deionized water. Following momentary drying under nitrogen flow, the stamps were placed onto the oxygen plasma-treated polyethylene substrates and weighed down with 500 g of mass. After 2 mins, the stamps were removed.

### Fluorescence imaging of patterned fluorescently labelled oligonucleotides

Baseline imaging of printed arrays was conducted at an exposure time of 1s using a Nikon Ti-2 inverted microscope. Images were captured at 4× and 10× magnifications. Stability testing involved imaging under the same parameters after 30 mins of immersed water wash at 220 RPM using a shaking incubator (VWR, Ontario, Canada). Food overlay imaging using romaine lettuce, ground beef, and whole chicken was done under the same parameters, but with 10 mm thick samples placed on the arrays.

### fNAP synthesis

A substrate strand possessing a fluorophore-quencher separated by a riboadenosine base (Keck Oligonucleotide Synthesis Facility, Yale University, Connecticut, United States) was enzymatically ligated to the 5’ terminal of a probe fragment with a 3’ amino modification (Integrated DNA Technologies, Iowa, United States). This was templated by a splint strand. In preparation for ligation, 4 nmol of the probe strand was first phosphorylated using excess adenosine triphosphate, 1× T4 polynucleotide kinase buffer A and 40 U T4 polynucleotide kinase in a 50 µL reaction at 37 °C for 30 minutes (Thermofisher Scientific, Ontario, Canada). Following phosphorylation, 4 nmol of the substrate strand and 4 nmol of the split strand were added to the reaction, then heated at 90 °C for 1 minute and cooled at room temperature for 5 minutes to anneal the fragments. The cooled ligation reaction was then diluted to 400 µl by addition of T4 DNA ligase buffer to 1×, 20 U T4 DNA ligase, and water (Thermofisher Scientific, Ontario, Canada). The ligation reaction was then incubated at room temperature for 1 hour and ethanol precipitated using 2.5 volumes of chilled ethanol, followed by centrifugation at 4 °C, 20000 × g for 20 minutes. The ligated product was purified on a 10%, 8 M urea polyacrylamide gel. The band corresponding to the ligated product was excised from the gel, crushed, and eluted in buffer (200 mM NaCl, 10 mM Tris pH 7.5, 1 mM EDTA; Thermofisher Scientific, Ontario, Canada). The elution supernatant containing the nucleic acid probe was then ethanol precipitated, followed by a final wash with 70% ethanol. The pellet was then air dried and resuspended in water.

### Inkjet-printed fNAP arrays

A print solution containing 3 μM nucleic acid probe, EDC, and NHS was prepared in MES buffer at a pH of 4.5 (Millipore Sigma, Ontario, Canada)^50^. A GeSIM Nanoplotter 2.0 (Radeberg, Germany) was used to deposit 12 nL droplets onto carbon dioxide plasma-treated polyethylene substrates. Each array was composed of 36 spots arranged in a 6 × 6 configuration. Each slide contained four arrays, ensuring replicates with all subsequent assays. Printed arrays were incubated at 70% humidity for 2 h to mediate covalent attachment. The sensors were then washed in deionized water for 30 mins at 220 RPM using a shaker set to 40 °C.

### Contact-printed fNAP arrays

A print solution containing 3 μM nucleic acid probe, EDC, and NHS was prepared in MES buffer at a pH of 4.5^50^. 10 μL of the solution was deposited onto polydimethylsiloxane stamps and incubated for 8 mins. Concurrently, glass slides wrapped with the target polyethylene substrate were carbon dioxide plasma-treated for three minutes. The solution was subsequently pipetted off, to permit control over residual volume present on the stamps. The stamps were then momentarily exposed to nitrogen flow, and then placed onto carbon dioxide plasma-treated substrates and weighed down with 500g of mass under 65% humidity. After two minutes, stamps were removed. The substrates were left under humidity for 30 mins before subsequent testing to permit covalent attachment.

### fNAP functionality testing

Inkjet and contact printed arrays were incubated with 0.5 M sodium hydroxide (Millipore Sigma, Texas, United States) as a positive test condition and deionized water as a negative test condition^21^. Arrays were imaged prior to incubation to establish baseline intensities. Arrays were then incubated with 200 μL of test solution for 2 h and subsequently imaged again. All imaging occurred at 1 s exposure using a Nikon Ti-2 inverted microscope. The visibility of positive state fNAPs when overlaid with food involved imaging with 10 mm samples of romaine lettuce, ground beef, and whole chicken placed on top of the arrays.

### Fluorescence quantification

Mean fluorescence intensities for the determination of the inherent fluorescence of target food products were calculated by the NIS Elements imaging software. SNR and CV values of oligonucleotide arrays both with and without overlaid romaine lettuce, ground beef, and whole chicken food backgrounds were calculated using ImageJ.

### Statistical analysis

All data in this work are presented as the mean across a minimum of triplicate measurements with all error bars representing the standard deviation across all measurements. A paired, 2-tailed t-test, CI 95% was used to analyze the SNRs of fNAP before and after the wash cycle (Figure 5b). One way ANOVA tests, CI 95%, were used to analyze the SNRs of the fNAP arrays before and after testing with buffer and NaOH (Figure 5d.i) and the positive and negative state fNAP arrays when overlaid with romaine lettuce, ground beef, and whole chicken samples (Figure 5f.i). *P* values greater than 0.05 were considered insignificant indicated by *ns* and *P* < 0.0001 was indicated by ****. All graph development and associated statistical analysis was completed using GraphPad Prism.

### Supplementary Information

Supplementary Information.

## Data Availability

All data are available in the main text, the supplementary materials, or provided upon request.
