# Supplementary material for: Comprehensive fluorescence profiles of contamination-prone foods applied to the design of microcontact-printed in situ functional oligonucleotide sensors
Source: Sci Rep. 2024 Apr 9;14:8277. doi: 10.1038/s41598-024-58698-0 (PMC11004136; doi:10.1038/s41598-024-58698-0)
Supplement: Supplementary file 1 — Supplementary Information. [file 41598_2024_58698_MOESM1_ESM.docx]

**Supplementary Information**

**Comprehensive Fluorescence Profiles of Contamination-prone Foods Applied to the Design of Microcontact-printed *in situ* Functional Oligonucleotide Sensors**

Shadman Khan^a^, Amid Shakeri^b^, Jonathan K. Monteiro^c^, Simrun Tariq^d^, Akansha Prasad^a^, Jimmy Gu^d^, Carlos D.M. Filipe^e^, Yingfu Li*^,d^, Tohid F. Didar*^,a,b^

^a^ School of Biomedical Engineering, McMaster University, 1280 Main Street West, Hamilton, ON L8S 4L8, Canada.

khans114@mcmaster.ca; prasaa4@mcmaster.ca; didart@mcmaster.ca

^b^ Department of Mechanical Engineering, McMaster University, 1280 Main Street West, Hamilton, ON L8S 4L7, Canada.

shakeria@mcmaster.ca; didar@mcmaster.ca

^c^ Department of Medicine, McMaster University, 1280 Main Street West, Hamilton, ON L8S 4K1, Canada.

montej2@mcmaster.ca

^d^ Department of Biochemistry and Biomedical Sciences, McMaster University, 1280 Main Street West, Hamilton, ON L8S 4K1, Canada.

tariqs10@mcmaster.ca; guj4@mcmaster.ca; liying@mcmaster.ca

^e^ Department of Chemical Engineering, McMaster University, 1280 Main Street West, Hamilton, ON L8S 4L7, Canada.

filipec@mcmaster.ca

^*^ Correspondence: Tohid Didar: [didart@mcmaster.ca](mailto:didart@mcmaster.ca), Yingfu Li: [liying@mcmaster.ca](mailto:liying@mcmaster.ca)

**Supplementary Table S1.** Fluorescently labelled single stranded oligonucleotides used in the study.

| **Probe** | **Sequence** |
| --- | --- |
| **PacBlue** | ACCAGTGCTCAGACA/PacBlue |
| **FAM** | AmC6/ACCAGTGCTCAGACA/36-FAM |
| **Cy3** | AmC6/ACCAGTGCTCAGACA/Cy3 |
| **Cy5** | AmC6/ACCAGTGCTCAGACA/Cy5 |


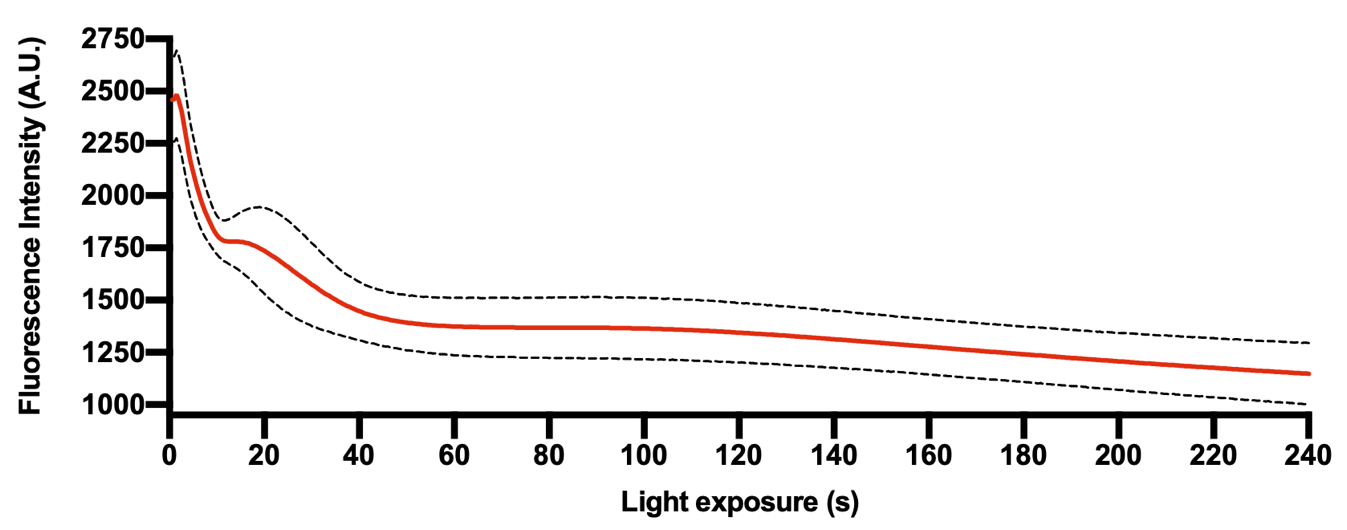

**Supplementary Figure S1.** Photobleaching of romaine lettuce under the Cy5 channel over four minutes.


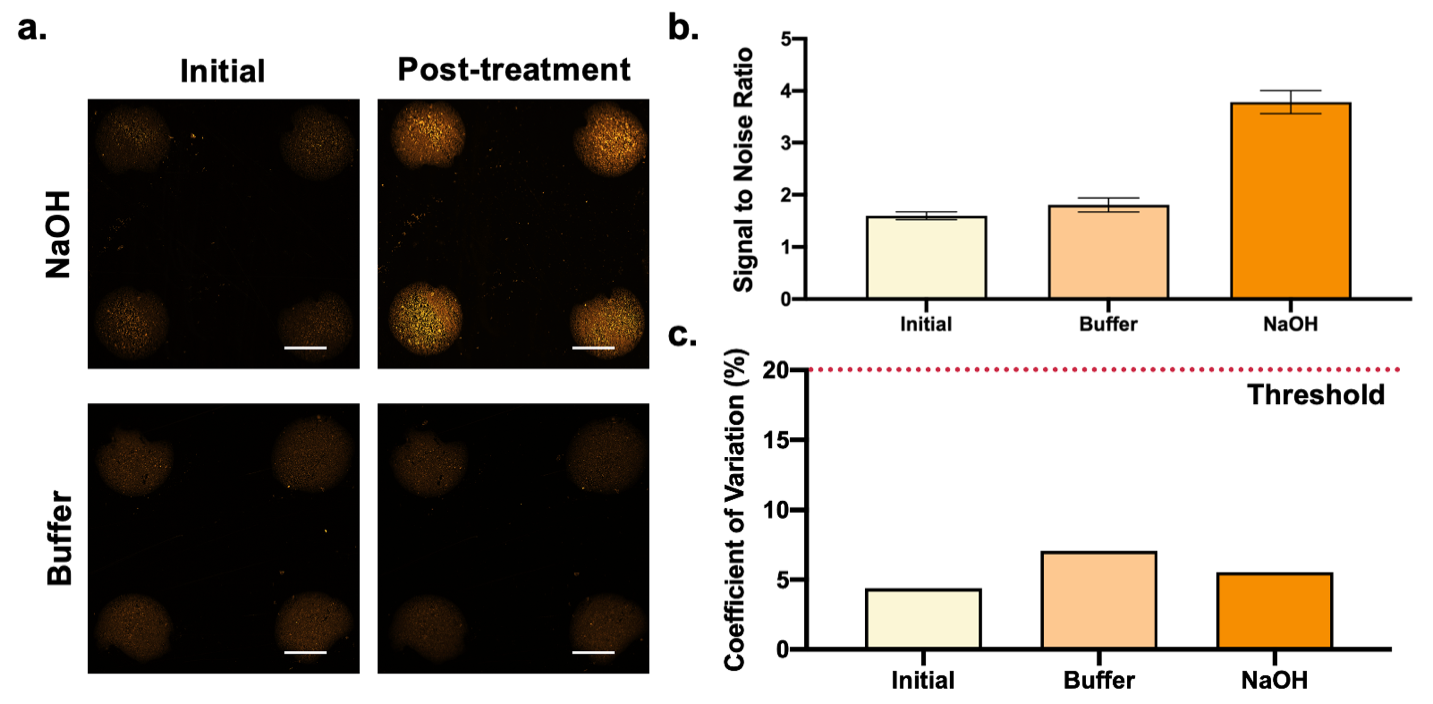
 **Supplementary Figure S2.** **Inkjet-printed RNA-cleaving fluorescent nucleic acid probe arrays.** (a) Fluorescence images before and after positive and negative control treatments. Scale bars indicate 200μm. (b-c) SNR and CV values of resultant arrays, respectively. Error bars represent standard deviation.

**Supplementary
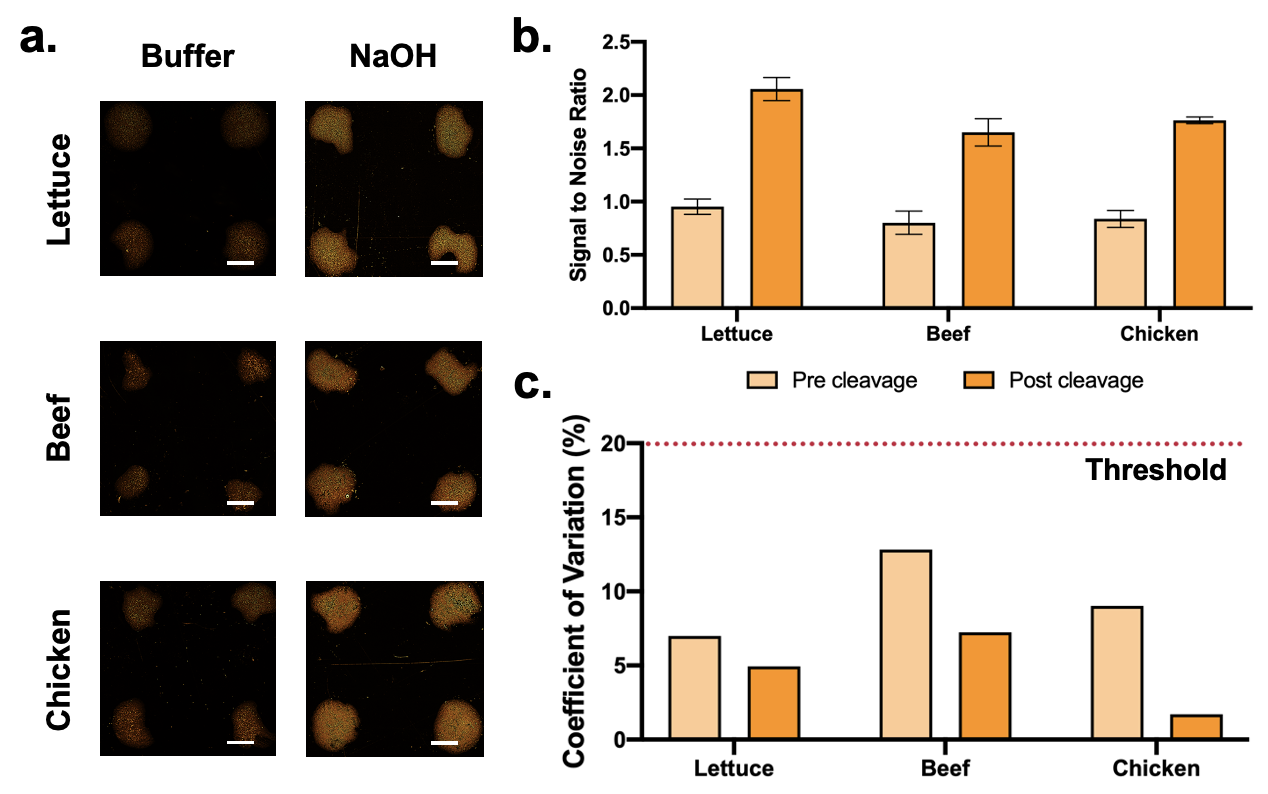
 Figure S3.** **Inkjet-printed RNA-cleaving fluorescent nucleic acid probe arrays overlaid with food products**. (a) Fluorescence images of positive and negative control-treated arrays with overlaid food samples. Scale bars indicate 200μm. (b-c) SNR and CV values of resultant arrays, respectively. Error bars represent standard deviation.
